# Non-Destructive Freshness Assessment of Atlantic Salmon (*Salmo salar*) via Hyperspectral Imaging and an SPA-Enhanced Transformer Framework

**DOI:** 10.3390/foods15040725

**Published:** 2026-02-15

**Authors:** Zhongquan Jiang, Yu Li, Mincheng Xie, Hanye Zhang, Haiyan Zhang, Guangxin Yang, Peng Wang, Tao Yuan, Xiaosheng Shen

**Affiliations:** 1 East China Sea Fisheries Research Institute, Chinese Academy of Fishery Sciences, Shanghai 200090, China; zhongquanj@sjtu.edu.cn (Z.J.);; 2 Key Laboratory of Environmental Health Impact Assessment of Emerging Contaminants, Ministry of Ecology and Environment, School of Environmental Science and Engineering, Shanghai Jiao Tong University, Shanghai 200240, China

**Keywords:** global attention mechanism, spectral non-linearity, food quality control, Successive Projections Algorithm (SPA), deep learning photonics, *Salmo salar*

## Abstract

Monitoring the freshness of *Salmo salar* within cold chain logistics is paramount for ensuring food safety. However, conventional physicochemical and microbiological assays are impeded by inherent limitations, including destructiveness and significant time latency, rendering them inadequate for the real-time, non-invasive inspection demands of modern industry. Here, we present a novel detection framework synergizing hyperspectral imaging (400–1000 nm) with the Transformer deep learning architecture. Through a rigorous comparative analysis of twelve preprocessing protocols and four feature wavelength selection algorithms (Lasso, Genetic Algorithm, Successive Projections Algorithm, and Random Frog), prediction models for Total Volatile Basic Nitrogen (TVB-N) and Total Viable Count (TVC) were established. Furthermore, the capacity of the Transformer to capture long-range spectral dependencies was systematically investigated. Experimental results demonstrate that the model integrating Savitzky-Golay (SG) smoothing with the Transformer yielded optimal performance across the full spectrum, achieving determination coefficients (R^2^) of 0.9716 and 0.9721 for the Prediction Sets of TVB-N and TVC, respectively. Following the extraction of 30 characteristic wavelengths via the Successive Projections Algorithm (SPA), the streamlined model retained exceptional predictive precision (R^2^ ≥ 0.95) while enhancing computational efficiency by a factor of approximately six. This study validates the superiority of attention-mechanism-based deep learning algorithms in hyperspectral data analysis. These findings provide a theoretical foundation and technical underpinning for the development of cost-effective, high-efficiency portable multispectral sensors, thereby facilitating the intelligent transformation of the aquatic product supply chain.

## 1. Introduction

Driven by the growing prevalence of health-conscious dietary patterns, the nutrient-dense Atlantic salmon (*Salmo salar*) has emerged as a strategic global economic commodity. Specifically, for the Atlantic salmon industry, market projections indicate a robust global trajectory, with the valuation expected to surge from USD 19.1 billion in 2024 to USD 44.4 billion by 2034, registering a Compound Annual Growth Rate (CAGR) of 8.8% [1]. This expansion is predominantly driven by the burgeoning demand for premium fillets in European and Asian markets, underscoring the necessity for advanced freshness monitoring to facilitate long-distance cold-chain trade. However, characterized by high lipid and moisture content, this perishable commodity exhibits acute sensitivity to temperature fluctuations. Psychrotrophic resilience in pathogens such as *Listeria monocytogenes* and *Salmonella* permits viability even under refrigeration, leading to recurrent food safety incidents that pose substantial risks to public health [2]. Consequently, the development of a real-time, precision-driven early warning system capable of monitoring freshness and microbial status is imperative for safeguarding public health and ensuring the industry’s sustainable trajectory.

Currently, Total Volatile Basic Nitrogen (TVB-N) and Total Viable Count (TVC) serve as the authoritative indices for freshness evaluation. The former quantifies alkaline byproducts resulting from the oxidative decomposition of proteins and non-protein nitrogen [3], while the latter constitutes the “gold standard” for assessing microbial load [4]. Despite their scientific validity, traditional physicochemical and microbiological assays are impeded by inherent limitations, including destructiveness and significant time latency—ranging from several hours for chemical analysis to 48–72 h for microbial culture. These constraints render them inadequate for modern cold chain logistics, which demand real-time, non-destructive, and comprehensive inspection capabilities [3,4]. There is, therefore, a critical industrial exigency for a detection modality that ensures comprehensive, real-time, and non-invasive assessment—a technological void that hyperspectral imaging (HSI) is uniquely poised to fill.

Hyperspectral Imaging (HSI) represents a synergistic convergence of spectroscopy and computer vision. Distinct from conventional RGB photography, which is limited to three broad channels (Red, Green, Blue), HSI systems capture spectral information across hundreds of narrow, contiguous bands within a continuous spectrum (e.g., 400–1000 nm). The resultant data structure is characterized as a three-dimensional “hypercube” [5]. For each spatial pixel on the salmon fillet, a complete spectral signature is acquired. Analogous to a “molecular fingerprint,” this signature records the absorption, reflection, and scattering properties of the constituent materials within that specific micro-region. This technology has demonstrated utility in the rapid assessment of meat freshness, as evidenced by studies utilizing HSI to quantify TVB-N and TVC levels in chicken breast [6].

The selection of HSI for this study is predicated on the strong physical correlation between the biochemical degradation of Atlantic salmon and its spectral characteristics. As demonstrated by Wu and Sun [7], hyperspectral signatures in the VIS and SW-NIR regions serve as a non-invasive proxy for microbial spoilage (TVC) and chemical decomposition (TVB-N). Specifically, the 400–1000 nm range captures the complex interplay between electronic transitions in heme pigments and the harmonic overtones of fundamental chemical bonds associated with spoilage metabolites. During storage, the complex interplay between biochemical degradation and chromatic evolution is driven by multiple pathways. While astaxanthin serves as a key protective antioxidant, the gradual depletion of endogenous antioxidant systems eventually leads to its oxidative degradation, which precipitates a loss of the characteristic vibrant orange-red pigmentation.

Concurrently, heme pigments undergo significant redox transitions; specifically, muscle myoglobin and hemoglobin shift from their oxygenated forms to metmyoglobin and methemoglobin, contributing to browning. This process is further exacerbated by lipid oxidation, whose secondary metabolites interact with these pigments to accelerate color loss. The electronic transitions of these pigment molecules induce specific shifts in absorption peaks within the visible spectrum; HSI detects these subtle chromatic evolutions with a sensitivity far exceeding human visual perception [8]. However, the richness of this high-dimensional data introduces substantial computational challenges. The spectral responses of adjacent bands exhibit high multicollinearity (e.g., information at 650 nm is virtually identical to 651 nm), which severely compromises the stability of traditional linear regression models [9]. Furthermore, the hypercube is laden with redundancy; many bands may contain only noise or artifacts arising from ambient lighting and surface scattering rather than informative features. The processing of hypercubes containing tens of thousands of pixels and hundreds of bands imposes a heavy computational burden, constraining the speed requisite for industrial on-line inspection [10]. Consequently, the extraction of robust features highly correlated with TVB-N and TVC from massive, redundant, and noisy spectral data, followed by the construction of high-precision prediction models, constitutes the core technical bottleneck addressed in this study.

To navigate this complex data landscape, spectral analysis algorithms have evolved from parsimonious linear models to sophisticated deep neural architectures. Early spectral analytics predominantly relied on Partial Least Squares Regression (PLSR), which effectively mitigates multicollinearity by projecting high-dimensional spectral data onto a reduced set of Latent Variables [11]. However, PLSR is fundamentally a linear construct, predicated on the strict adherence of spectral absorbance to the Beer-Lambert Law. In heterogeneous biological matrices such as *Salmo salar*, light undergoes not only absorption but also stochastic multiple scattering. The spoilage process entails a cascade of complex physicochemical alterations—including protein denaturation, lipid oxidation, and biofilm formation—rendering the spectral response inherently non-linear. Linear models fail to resolve these intricate non-linearities, thereby creating a bottleneck in prediction accuracy [12].

In response, the advent of deep learning introduced Convolutional Neural Networks (CNNs) to spectral analysis. 1D-CNNs deploy convolutional kernels to traverse the spectral sequence, automatically extracting local morphological features such as peak shapes, slopes, and inflection points [13]. Nevertheless, the intrinsic mechanism of a CNN is bound by its Local Receptive Field; a single kernel typically spans only 3–7 bands. While stacking network layers can expand this field, CNNs remain inefficient at capturing long-range dependencies across the full spectrum [14]. This study is predicated on the hypothesis that if the biochemical and microbial spoilage of Atlantic salmon muscle generates non-linear and long-range dependent spectral patterns in the 400–1000 nm range, then architectures based on global attention (Transformer) will model these patterns more accurately than linear or locally constrained convolutional models.

To transcend the limitations of convolutional architectures, this study incorporates the Transformer architecture, which has revolutionized Natural Language Processing (NLP). Eschewing recurrence and convolution, the Transformer relies entirely on the Self-Attention Mechanism [15]. Its cardinal advantage lies in its capacity to model long-range dependencies: through self-attention, every point (band) in the sequence computes a correlation weight with every other point simultaneously [16]. Furthermore, the model exhibits dynamic adaptivity. For distinct samples, the Transformer dynamically allocates attention weights; for instance, if spoilage manifests primarily as moisture loss, the model automatically assigns higher weights to water-absorption bands, whereas it shifts focus to lipid-associated bands if oxidation is dominant. This adaptive capability stands in sharp contrast to the rigid, fixed-weight kernels of CNNs [17]. However, the Transformer architecture is not without its own constraints.

Notwithstanding its formidable performance, the Transformer architecture is constrained by a computational complexity that scales quadratically with sequence length O(N2). Ingesting raw, full-spectrum data not only incurs a prohibitive computational cost but also exacerbates the risk of overfitting due to high-dimensional noise. Consequently, rigorous feature wavelength extraction is a prerequisite for achieving model lightweighting and facilitating industrial deployment.

Building upon a comparative analysis of Lasso, Genetic Algorithms (GA), and Random Frog, this study prioritizes the Successive Projections Algorithm (SPA). SPA functions as a forward variable selection technique designed to minimize collinearity through vector projection operations. It distills the most informative, low-redundancy bands from the massive spectral continuum [18]. By integrating SPA with the Transformer, we aim to engineer a “parsimonious yet robust” architecture—one that retains the Transformer’s global modeling capabilities while excising noise interference through SPA, thereby significantly enhancing computational efficiency. This approach also provides the theoretical underpinning for the future development of low-cost multispectral sensors based on discrete wavelengths [10].

Predicated on this critical review and the theoretical superiority of global attention mechanisms in resolution of complex spectral signatures, this study aims to resolve the challenge of rapid, non-destructive freshness assessment in Atlantic salmon by coupling hyperspectral imaging with an optimized, hypothesis-driven Transformer framework. By presenting a solution rooted in “Deep Learning Photonics,” we seek to drive a paradigm shift in the food industry toward intelligent, precision-driven manufacturing.

## 2. Materials and Methods

### 2.1. Experimental Materials

“Specimens of Atlantic salmon, originating from Norwegian aquaculture facilities, were procured from Hema Fresh Supermarket (Shanghai, China) on 28 March 2025. The selection of Atlantic salmon from a specific origin and batch was a deliberate experimental design to minimize confounding variables, such as aquaculture environment and fishing seasonality, thereby allowing for a more rigorous validation of the SPA-Transformer framework’s core efficacy in feature selection and freshness prediction. The samples were from the 4–5 kg size class and were harvested on 22 March 2025. Following a 6-day international cold-chain transit via air freight, cold-chain integrity was objectively confirmed via temperature logging, ensuring a stable thermal profile of 0–2 °C prior to procurement. All samples were derived from the mid-dorsal region, vacuum-sealed in food-grade polyethylene packaging with a net mass of 180 ± 5 g per unit.” Visual inspection confirmed that the fillets were devoid of mechanical damage or ecchymosis and exhibited a vibrant orange-red pigmentation, in strict compliance with the National Food Safety Standard for Fresh and Frozen Animal Aquatic Products [19]. To maintain thermal stability during transit to the laboratory, samples were housed in insulated polystyrene containers fortified with cryogenic ice packs. Upon arrival, 96 sample units were randomly stratified into two distinct thermal cohorts and stored in precision-controlled environmental chambers at 4 ± 0.5 °C and 8 ± 0.5 °C, respectively. The selection of these temperatures was predicated on simulating representative commercial scenarios: 4 °C represents the standard refrigeration temperature widely adopted in global cold-chain logistics and retail for iced salmon, reflecting ideal storage practices. Conversely, the 8 °C cohort was established to simulate common temperature fluctuations or localized cold-chain failures often encountered in real-world logistics, thereby facilitating the construction of an accelerated spoilage model to evaluate the predictive robustness of the SPA-Transformer framework under varying degradation rates. In this study, an ‘independent sample’ is defined as a standardized portion of dorsal muscle derived from these units. Sampling was conducted at eight discrete time points: 0, 1, 2, 3, 5, 7, 9, and 11 days. Subsampling procedures were performed within a laminar flow cabinet to ensure sterility. Following the removal of vacuum packaging, fillets were aseptically peeled and vertically cut along the direction of muscle segments using ethanol-sterilized (75% *v*/*v*) stainless steel instrumentation into standardized rectangular prisms (6 cm × 3 cm × 2 cm). This standardized preparation aimed to eliminate spectral background noise and artifacts arising from fish skin connective tissues or irregular mechanical damage, ensuring that the signals captured by the HSI system predominantly reflected the biochemical degradation of the muscle tissue. This balanced experimental design (2 temperatures × 8 time points × 30 replicates) resulted in a total of 480 independent samples (N = 480), providing a robust statistical basis for subsequent analysis.

### 2.2. Experimental Instrumentation and Methods

#### 2.2.1. Hyperspectral Image Acquisition and Calibration

Hyperspectral data acquisition was performed using a portable field hyperspectral imaging system (GaiaField-V10E, Jiangsu Dualix Spectral Imaging Technology Co., Ltd., Wuxi, China). The system operational parameters were calibrated as follows: an exposure time of 7.9 ms, a gain setting of 2, a push-broom scanning speed of 9 s/cube, and a spatial resolution of 0.1 mm/pixel. Illumination was provided by two 300 W tungsten lamps (Tianjin Yaohua Media Co., Ltd., Tianjin, China), mounted symmetrically at a 45° angle relative to the sample stage. The working distance from the focal lens to the specimen surface was fixed at 58 cm. All acquisition procedures were conducted within a light-tight darkroom to mitigate interference from ambient stray light.

**Figure 1:**
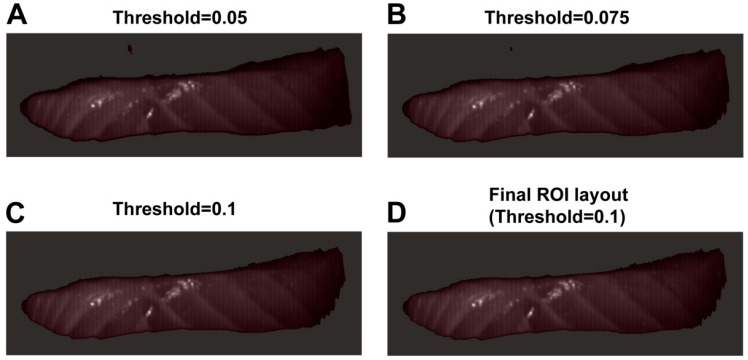
Comparison of ROI extraction using different threshold values in hyperspectral imaging. (**A**) Threshold = 0.05. (**B**) Threshold = 0.075. (**C**) Threshold = 0.1. (**D**) Final ROI layout with threshold = 0.1.

During the experimental phase, to comprehensively capture the surface spectral characteristics of each independent sample, bilateral scanning (dorsal and ventral surfaces) was performed (Figure 1). Consequently, each of the 480 samples yielded two distinct hyperspectral cubes, aggregating to a total dataset of 960 valid hyperspectral images. Data acquisition and system control were orchestrated via the SpectraVIEW software package (Version 1.2). To correct for heterogeneous illumination and sensor dark current noise, black-and-white radiometric calibration was performed prior to each acquisition sequence. This involved capturing a white reference using a Polytetrafluoroethylene (PTFE) standard board (~100% reflectance) and a dark reference with the lens cap secured (~0% reflectance). The reflectance correction was calculated according to the following equation:

$$Rc=\frac{R_{0}-B}{W-B}\times 100\%$$

where Rc denotes the calibrated hyperspectral image (relative reflectance), R_0_ represents the raw spectral image acquired from the sample, B corresponds to the dark reference image, and W refers to the white reference image.

#### 2.2.2. Determination of Physicochemical and Microbiological Indices: Determination of Total Volatile Basic Nitrogen (TVB-N)

The Total Volatile Basic Nitrogen (TVB-N) content was quantified in strict accordance with Method I (semimicro nitrogen determination) specified in the National Food Safety Standard for Determination of Volatile Basic Nitrogen in Foods [20]. Analyses were performed utilizing a KDN-CZ intelligent nitrogen analyzer (Shanghai Xijin Biological Group Co., Ltd., Shanghai, China). For each specimen, assays were conducted in duplicate, and the arithmetic mean was recorded. The final TVB-N concentration is expressed in mg/100 g; The Total Viable Count (TVC) was assessed using the 3M™ Petrifilm™ Aerobic Count Plate method (3M Company, St. Paul, MN, USA) [21]. This ‘Rapid Determination’ designation refers to the streamlined sample preparation and inoculation process, which offers superior operational efficiency compared to traditional pour plate assays. Inoculated test sheets were incubated in a thermostatic chamber at 30 ± 1 °C for 72 ± 3 h, in strict accordance with standard procedures for psychrotrophic organisms in fish products [22,23]. Following incubation, colony enumeration was performed with a detection limit of 1 CFU/g. Results were averaged and transformed into the base-10 logarithm (log_10_), expressed as lg CFU/g.

Given the complex background interference inherent in raw hyperspectral imagery, a rigorous image segmentation protocol was implemented to mitigate its impact on feature extraction precision. Data processing workflows were executed within a Python 3.9 environment, utilizing the spectral library for data ingestion and the matplotlib library to facilitate threshold analysis. To ensure robust background suppression independent of specific spectral fluctuations, the threshold segmentation was performed on the mean grayscale image calculated across the full spectral range (400–1000 nm). Following a comparative evaluation of segmentation performance across three grayscale thresholds—0.05, 0.075, and 0.1 (refer to the “Results and Discussion” Section 3 and Section 4 for detailed comparative analysis)—a threshold value of 0.1 was selected as optimal. To ensure the integrity of the binary mask and eliminate isolated noise pixels, a morphological opening operation (using a 3 × 3 square structuring element) was applied to the resulting ROI mask. During patch generation, any window exceeding the ROI mask boundary or containing more than 5% background pixels was categorized as an exception and systematically discarded. After discarding these peripheral and low-purity patches, a final repository of 6342 standardized patches was obtained.

To address dimensional inconsistencies arising from morphological variations in salmon samples and to adhere to the input specifications of deep learning architectures, a dataset construction strategy employing a sliding window technique was adopted. The ROI of the 960 images was traversed and cropped using a non-overlapping sliding window (64 × 64 pixels). The 64 × 64 size was selected to preserve essential spatial features, such as muscle texture and fat distribution, while avoiding the sharp increase in computational complexity associated with excessively large input dimensions. This specific configuration allows for the generation of a 4096-token sequence, which is optimally suited for the joint “spatial-spectral” analysis requirements of *Salmo salar* hyperspectral images. After discarding peripheral patches containing background noise, a final repository of 6342 standardized patches was obtained. Regarding label assignment, TVB-N and TVC values were determined for each of the 480 independent samples immediately following imaging. All patches subsequently derived from a specific sample inherited the identical physicochemical and microbiological labels of their source sample. This one-to-many label inheritance scheme ensures a strict stoichiometric correspondence between spectral features and freshness indices while significantly enriching the feature diversity for model training. To ensure a rigorous evaluation and prevent data leakage, the dataset partitioning was executed at the individual sample level rather than the patch level. While 6342 patches were generated via the sliding window method to capture spatial heterogeneity, the division into training, validation, and prediction sets was strictly executed at the individual biological level based on the 480 source original fillets (N = 480). All patches derived from the same independent sample were strictly grouped within the same subset, ensuring that the model was tested on spectral signatures from entirely independent biological units. Following a 70:15:15 ratio, the Training Set comprised 336 fillets (approx. 4440 patches), the Validation Set included 72 fillets (951 patches), and the Prediction Set contained 72 fillets (951 patches). This individual-based stratification strategy effectively mitigates the risk of over-fitting to localized sample artifacts and substantiates the model’s robustness. This patch-based partitioning strategy is predicated on the inherent spatial heterogeneity of Atlantic salmon muscle tissue, ensuring that each non-overlapping patch represents a distinct biochemical micro-environment rather than redundant global information, thereby effectively mitigating the risk of data leakage while maximizing the model’s exposure to localized degradation patterns. Specifically, the Training Set (N = 4440) was utilized for parameter fitting, the Validation Set (N = 951) for optimization, and the Prediction Set (N = 951) for independent testing. In this framework, patches derived from the same fillet were considered independent local feature units. This approach is predicated on the inherent spatial heterogeneity of Atlantic salmon muscle—such as variations in fiber density and moisture distribution—which ensures that these patches represent distinct biochemical micro-environments rather than redundant information. Specifically, the Training Set (N = 4440) was utilized for model parameter fitting; the Validation Set (N = 951) was employed for hyperparameter optimization and overfitting surveillance; and the Prediction Set (N = 951) served as an independent prediction set to verify actual detection capabilities on unseen samples.

To mitigate physical artifacts inherent in the data acquisition process—specifically instrumental noise, surface scattering effects, and baseline drift—and to augment the spectral signal-to-noise ratio (SNR), a comparative evaluation of twelve distinct preprocessing algorithms was conducted. The specific methodologies encompassed: Multiplicative Scatter Correction (MSC), Savitzky-Golay smoothing (SG), Standard Normal Variate (SNV) transformation, Moving Average filtering, First and Second Derivatives, Wavelet Transform, Mean Centering, Standardization, Baseline Correction, Min-Max Normalization, and Vector Normalization. In the preliminary screening stage, the spectral data processed by each protocol were quantitatively evaluated using three core indicators: SNR, coefficient of variation (CV), and baseline offset. Based on these metrics, four protocols with superior comprehensive performance—Baseline correction, SNV, SG, and Min-Max normalization—were selected for subsequent modeling. To ensure the objectivity of the comparison, this study strictly controlled variable consistency; all four protocols utilized the identical data partitioning (70%:15%:15%) and the same hyperparameter configurations during model training.

#### 2.2.3. Feature Wavelength Extraction

In light of the high dimensionality and substantial redundancy intrinsic to hyperspectral data, full-spectrum modeling is prone to excessive computational burdens and model overfitting. To excise irrelevant variables and screen for feature wavelengths exhibiting high correlation with TVB-N and TVC variations, this study conducted a comparative analysis of four distinct feature selection algorithms:

Lasso (Least Absolute Shrinkage and Selection Operator): Utilizing L1 regularization, this method compresses the coefficients of non-critical variables to zero, thereby achieving sparse feature selection.

Genetic Algorithm (GA): Predicated on the principles of biological evolution, the GA iteratively searches for the global optimal feature combination through selection, crossover, and mutation operations.

Successive Projections Algorithm (SPA): This algorithm employs vector projection analysis to eliminate collinearity among wavelengths, isolating a subset of wavelengths containing minimal redundant information. In this study, the candidate pool for SPA comprised all 158 spectral bands. The algorithm utilized a multiple linear regression (MLR) evaluator to optimize the selection process, with the minimization of the Root Mean Square Error of Calibration (RMSEC) serving as the objective function. The iterative procedure was terminated when the RMSEC reached its minimum value, ensuring an optimal balance between model parsimony and predictive accuracy.

Random Frog (RF): Simulating biological foraging behavior, this algorithm performs an efficient search within the solution space utilizing Reversible Jump Markov Chain Monte Carlo (RJMCMC) strategies.

#### 2.2.4. Machine Learning Models

To facilitate the conceptual validation of the proposed hypothesis, a comprehensive modeling framework encompassing linear, locally constrained, and global attention architectures was established. To quantitatively evaluate the impact of the global receptive field, the comparative performance across these distinct mathematical approaches was assessed not only through absolute metrics but also through the relative gains in predictive precision (R^2^) and error reduction (RMSE) achieved by the Transformer over linear baselines (MLR and PLSR). This quantitative comparison serves as a direct test to evaluate whether the global attention mechanism provides a statistically significant inherent advantage in resolving the complex, non-linear, and long-range dependent spectral signatures of salmon spoilage. Four representative algorithms were selected to serve as the baseline for this conceptual test:

Multiple Linear Regression (MLR): Serving as the benchmark model, MLR was utilized to evaluate the linear associations between spectral features and physicochemical indices.

Partial Least Squares Regression (PLSR): By extracting Latent Variables (LVs), PLSR effectively resolves the issue of severe multicollinearity inherent in hyperspectral data.

Support Vector Machine (SVM): Leveraging kernel function mapping, SVM demonstrates superior generalization capabilities in small-sample and non-linear regression tasks.

Random Forest (RF): Rooted in ensemble learning theory, this model exhibits robust resilience against outliers and provides an intrinsic assessment of feature importance.

**Deep Learning Model**: To further extract deep-level features from the high-dimensional data, we utilized a MLP and a Transformer architecture. Given that hyperspectral signatures are inherently sequential, the Transformer’s core self-attention mechanism is uniquely positioned to capture long-range dependencies between biochemical signals (e.g., amine accumulation) and the moisture-driven scattering background [7]. To transition from a narrative to a quantitative spectral interpretation, a visualization protocol for attention importance was implemented. Specifically, attention weights were extracted from the 8-head Multi-Head Self-Attention (MSA) layers to generate per-band attention maps. By aggregating weights across heads and spatial tokens, the model provides quantitative evidence of its prioritization of specific spectral regions. This allows for an objective verification of whether the Transformer resolves high-dimensional dependencies by focusing on known biochemical vibrational modes rather than overfitting to stochastic background interference.

**Proposed Transformer Architecture Design**: Standardized patches (64 × 64 pixels × 158 bands) were flattened into sequences of 4096 tokens. The input tensor (B × 4096 × 158) was mapped via a linear embedding layer to a 256-dimensional hidden space. The architecture comprises an Input Projection module (Linear layer, LayerNorm, GELU, and Dropout), followed by Positional Encoding to retain spatial order information. The core feature extractor consists of three Transformer Encoder Layers, each utilizing an 8-head MSA mechanism and a Feed-Forward Network to capture correlated features across spectral and spatial dimensions. After Global Average Pooling, a three-stage cascaded linear regression head (128 → 64 → 32 → 1) outputs the predicted values. A ReLU activation function was applied to the final layer to enforce physical non-negativity constraints on TVB-N and TVC predictions, ensuring alignment with real-world food safety detection limits.

#### 2.2.5. Model Implementation and Training Protocol

Development Environment Configuration: Computational implementation and data analysis were executed within the PyCharm 2025.3 (Professional Edition) Integrated Development Environment (IDE) using the Python 3.9 programming language. Deep learning architectures were constructed leveraging the PyTorch v2.9.0 framework. To ensure a reproducible benchmark for computational efficiency, all models were evaluated on a standardized workstation equipped with an Intel (R) Core (TM) i5-14600KF CPU and an NVIDIA GeForce RTX 4070 SUPER GPU, operating on Windows 11 Professional with Microsoft Visual C++ 2019 Redistributable (x64) dependencies. Data standardization was performed utilizing the StandardScaler module from the Scikit-Learn library, while visual analytics were generated using a combination of the Matplotlib (v3.10.0) and Plotly (v6.0.0) libraries.

Hyperparameter Configuration and Training Protocol: Model optimization was performed using the AdamW algorithm, initialized with a learning rate of 1 × 10^−4^ and a weight decay coefficient of 5 × 10^−5^. The Mean Squared Error (MSELoss) served as the objective function for loss minimization. To facilitate optimal convergence, a ReduceLROnPlateau learning rate scheduling strategy was implemented. This mechanism applied a decay factor of 0.5 if the validation loss failed to decrease over a persistence window (patience) of 15 epochs, with a lower bound threshold set at 5 × 10^−7^. The maximum training duration was established at 200 epochs. To mitigate the risk of overfitting, an Early Stopping mechanism was incorporated, designed to automatically terminate the training process if no improvement in validation loss was observed for 30 consecutive epochs.

## 3. Results

### 3.1. Spectral Characteristics of Salmon Samples

**Figure 2:**
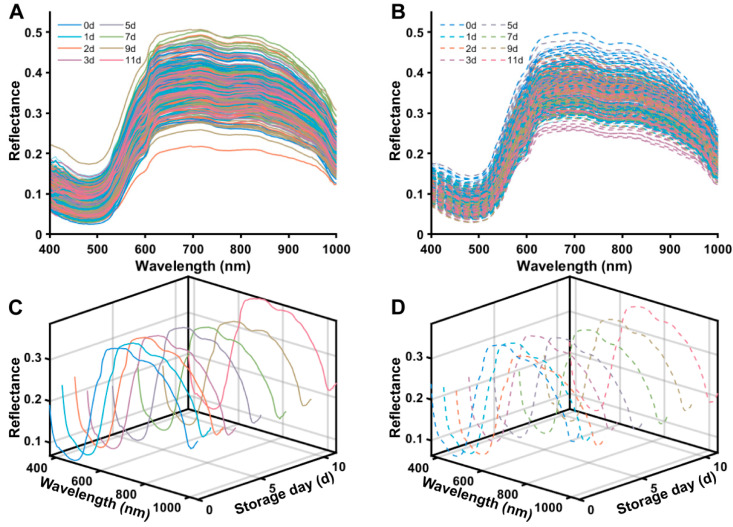
Spectral evolution of salmon samples during chilled storage at 4 °C and 8 °C. (**A**) Raw spectral reflectance at 4 °C; (**B**) Raw spectral reflectance at 8 °C; (**C**) Average reflectance at 4 °C and (**D**) at 8 °C for days 0, 1, 3, 5, 7, 9, and 11.

Figure 2 illustrates the mean spectral reflectance profiles of *Salmo salar* specimens subjected to distinct storage regimes (4 °C and 8 °C) over the experimental duration. In general, the peak reflectance across the full spectral range (400–1000 nm) remained below 0.5 for all samples, with reflectance intensity in the near-infrared (NIR) region consistently exceeding that of the visible spectrum. The spectral signatures exhibited a uniform morphological trend: a pronounced absorption trough within the 400–500 nm band, followed by a maximum peak in the 600–700 nm interval, and a subsequent gradual decline across the 700–1000 nm range.

As the storage period extended from day 0 to day 11, a global downward shift in spectral reflectance was observed, accompanied by a progressive increase in inter-curve dispersion over time. A comparative analysis of spectral evolution under different thermal conditions (Figure 2C,D) revealed that both the magnitude and rate of spectral alteration in the 8 °C cohort were significantly more pronounced than those in the 4 °C group. Specifically, within the short-wave band (400–500 nm), the absorption trough in the 8 °C group deepened more precipitously with storage time. Concurrently, in the medium-to-long wave band (550–750 nm), the peak attenuation was both more rapid and substantial in the 8 °C cohort. Furthermore, spectral deviation from fresh samples (Day 0) during the late storage phase was markedly higher at 8 °C compared to 4 °C, indicating that elevated temperatures induced more significant perturbations in spectral characteristics.

### 3.2. Analysis of TVB-N and TVC Determination Results

**Figure 3:**
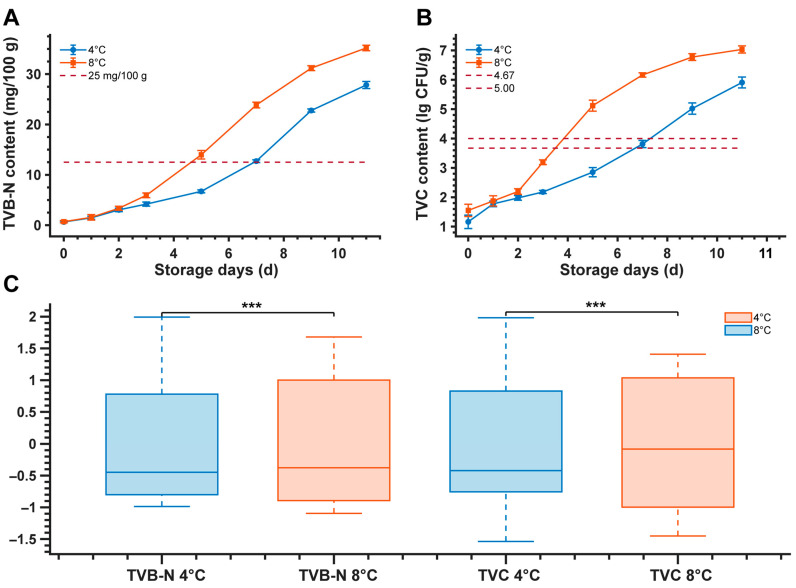
Time-course of TVB-N and TVC at 4 °C and 8 °C. (**A**) TVB-N (mg/100 g) and (**B**) TVC (lg CFU/g) with mean ± SD (*n* = 30). Red dashed lines indicate thresholds. (**C**) Temperature comparison: boxes represent IQR, whiskers extend to 1.5 × IQR. Y-axis values are z-scored; *p*-values and stars (***) are based on original data.

Figure 3 depicts the temporal evolution of TVB-N and TVC levels in salmon samples under different refrigeration temperatures (4 °C and 8 °C). As storage time progressed, TVB-N content in both groups exhibited a continuous upward trajectory (Figure 3A). Over the 11-day storage period, TVB-N values in the 4 °C group escalated from 1.29 mg/100 g to 55.67 mg/100 g, whereas the 8 °C group demonstrated a rapid surge from 1.40 mg/100 g to 70.38 mg/100 g, with the growth rate in the 8 °C cohort being significantly superior to that of the 4 °C group. Relative to the limit standard stipulated by GB 10136-2015 (25 mg/100 g), the 8 °C and 4 °C groups exceeded this threshold on day 5 and day 7, respectively [24].

TVC displayed a parallel growth pattern (Figure 3B): TVC values in the 4 °C group increased from an initial 2.16 lg CFU/g to 6.91 lg CFU/g, while the 8 °C group rose from 2.56 lg CFU/g to 8.03 lg CFU/g. With reference to the limit for ready-to-eat raw animal aquatic products (4.67 lg CFU/g), the 8 °C group surpassed the limit on day 4, and the 4 °C group on day 7. When applying the limit for marine fish (5.0 lg CFU/g), the violation times were day 4 (8 °C) and day 8 (4 °C), respectively. Furthermore, Pearson correlation analysis (Figure 3C) indicated a highly significant positive linear correlation between TVB-N and TVC values. To control for temperature as a confounding variable and specifically evaluate the model’s generalization across different thermal conditions, a stratified analysis was performed (n = 240 per cohort). The results yielded coefficients of determination (R^2^) exceeding 0.90 (*p* < 0.0001) for both the 4 °C and 8 °C groups independently. This suggests a high degree of consistency in the evolutionary trends of these indices during storage and confirms that the model effectively generalizes across varying thermal regimes, capturing spoilage-specific spectral signatures rather than temperature-dependent artifacts.

### 3.3. Efficacy of Spectral Data Preprocessing

**Figure 4:**
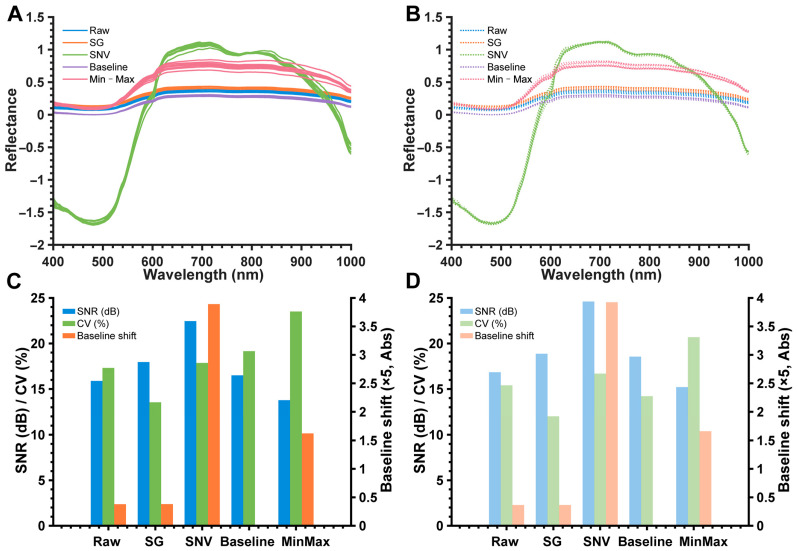
Comparison of spectral preprocessing methods for salmon samples at 4 °C and 8 °C. (**A**) Average raw and preprocessed spectral curves (Baseline, SNV, SG, Min-Max normalization) for 4 °C. (**B**) Average raw and preprocessed spectral curves for 8 °C. (**C**) Comparison of signal-to-noise ratio (SNR), coefficient of variation (CV), and baseline shift for different preprocessing methods at 4 °C. (**D**) Comparison of SNR, CV, and baseline shift at 8 °C.

A comprehensive pre-screening of the twelve initial preprocessing protocols was conducted using consistent quantitative criteria, including SNR, CV, and baseline offset (Figure 4). In terms of smoothing and denoising performance, SG Smoothing exhibited significantly higher smoothness compared to Wavelet Transform and Moving Average Filtering. SG Smoothing effectively suppressed random noise while fully preserving the peak shapes of spectral characteristics. Although First-Derivative and Second-Derivative methods highlighted spectral peak differences, they amplified baseline noise and caused severe overall fluctuations.

Regarding baseline optimization, Baseline Correction demonstrated markedly better baseline flatness than Mean Centering or Standardization. This method effectively eliminated background signal interference, facilitating the identification of characteristic peaks. For scattering and intensity normalization, SNV transformation outperformed Multiplicative Scatter Correction (MSC) and Vector Normalization by significantly reducing sample intensity differences. While Vector Normalization excessively compressed characteristic peak intensities, Min-Max Normalization achieved a unified magnitude while preserving relative peak intensity for subsequent analysis. Consequently, the pipeline integrating SG, SNV, Baseline Correction, and Min-Max Normalization was selected as the superior subset for subsequent modeling.

Regarding SNR, SNV transformation achieved the highest values (approx. 22.5 dB and 24.8 dB for 4 °C and 8 °C groups, respectively), whereas SG smoothing yielded a moderate SNR of approx. 18–19 dB. However, SNV processing induced a substantial baseline offset exceeding 3.0, which can introduce artifacts into high-dimensional modeling. In contrast, SG smoothing demonstrated the lowest data dispersion (CV of 12.0–13.5%) and ranking second in SNR and baseline stability. This comprehensive balance across SNR, CV, and baseline integrity identifies SG as the most robust candidate for maintaining spectral feature fidelity while minimizing stochastic interference.

### 3.4. Performance Evaluation of Prediction Models

Table 1 presents a detailed comparative analysis of the predictive performance of Transformer, PLSR, and MLR models for TVB-N and TVC content in salmon under various spectral preprocessing conditions. Notably, while SVM, RF, and MLP were also systematically trained and validated, their predictive outcomes exhibited a random distribution pattern and failed to establish any effective correlation with the freshness indicators. Consequently, to maintain the conciseness of the manuscript and prioritize the comparison of high-performing core models, these ineffective results were omitted from the final table. Following the overall trend, the Transformer architecture exhibited significantly superior predictive metrics compared to the PLSR (the best-performing linear baseline) and MLR models. This performance gap—characterized by a quantitative gain in $R^{2}$ of approximately 0.14 over PLSR—provides the first empirical evidence supporting our hypothesis regarding the non-linearity of spoilage spectra. Specifically, for TVB-N prediction, the Transformer’s prediction set $R^{2}$ reached 0.9716, whereas linear models remained constrained below 0.837. A paired *t*-test on the prediction residuals confirmed that the reduction in RMSE achieved by the Transformer was statistically significant (*p* < 0.05), providing explicit evidence for the model’s superior performance. This substantial improvement demonstrates that the global attention mechanism effectively captures high-dimensional variance that remains unresolved by traditional linear mapping.

In contrast, the Transformer model demonstrated superior fitting precision. Notably, when coupled with the Savitzky-Golay (SG) filter, the model achieved optimal comprehensive performance across both validation and prediction sets. For TVB-N prediction, the validation and prediction set R^2^ values reached 0.9728 and 0.9716, with RMSEs of 2.0275 and 3.0113, respectively. Similarly, for TVC prediction, the validation and prediction set R^2^ values were 0.9735 and 0.9721, with RMSEs as low as 0.3254 and 0.3306. Although the Transformer model based on SNV preprocessing yielded the highest fitting degree on the training set (TVB-N $R^{2}$ = 0.9941, TVC $R^{2}$ = 0.9929), the SG smoothing treatment conferred greater robustness on the independent prediction set. The minimal performance divergence between the training set and the prediction set (which integrates samples from both 4 °C and 8 °C storage) confirms that the synergy of the Transformer architecture and the SG smoothing algorithm effectively preserves the model’s generalization capability on unseen samples across different thermal cohorts. By training on a diverse temperature gradient, the SPA-Transformer is forced to prioritize long-range spectral dependencies that remain stable across different spoilage velocities, thereby ensuring its reliability for real-world cold-chain monitoring.

**Figure 5:**
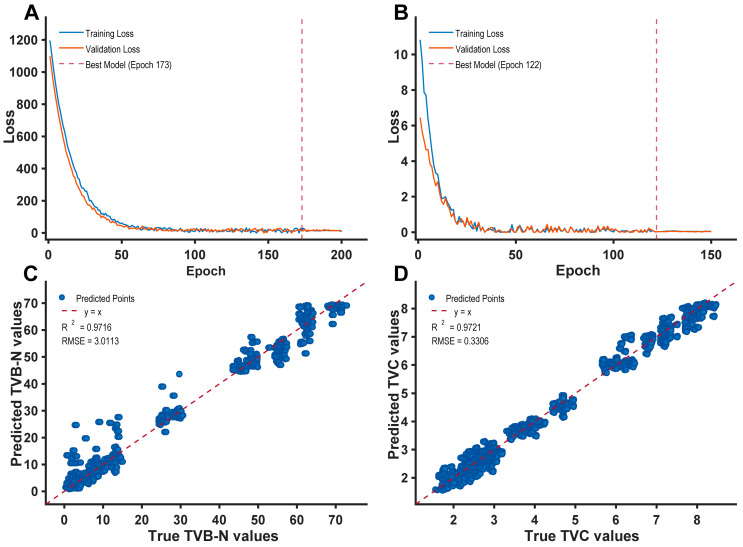
Prediction results using SG filter + Transformer model. (**A**) Training and validation loss for TVB-N. (**B**) Training and validation loss for TVC. (**C**) Predicted vs. true TVB-N values. (**D**) Predicted vs. true TVC values.

Figure 5 provides a visual representation of the convergence dynamics during the training phase and the performance efficacy on the independent prediction set for the Transformer model processed via SG smoothing. Inspection of the scatter plots (Figure 5C,D) reveals that the vast majority of predicted data points are tightly clustered along the diagonal identity line (y = x), signifying a high degree of concordance between predicted and actual values.

Quantitative metrics substantiate this observation: the model achieved a Coefficient of Determination (R^2^) of 0.9716 and a RMSE of 3.0113 for the prediction of TVB-N content. The predictive performance for TVC content was even more superior, yielding a prediction set R^2^ as high as 0.9721 and an RMSE as low as 0.3306. A comparative visual assessment indicates that the aggregation of TVC data points along the reference line is tighter, with significantly diminished deviation and dispersion compared to TVB-N. This distinction underscores the model’s exceptional fitting precision and stability, particularly in the prediction of microbial indices.

### 3.5. Performance Evaluation of Model Simplification

Cross-comparative analysis of various preprocessing methodologies and modeling frameworks establishes that the SG filter + Transformer configuration emerged as the optimal strategy. This combination demonstrated the highest accuracy in predicting salmon TVB-N and TVC values, outperforming other combinations in terms of both fitting efficacy and predictive stability. However, the inherent structural complexity of the Transformer architecture imposes substantial computational overhead and significant operation latency. These limitations inevitably constrain its efficient deployment in real-world, high-throughput detection scenarios.

To reconcile the trade-off between predictive precision and computational efficiency, this study undertook a simplification and optimization of the Transformer model, utilizing spectral data preprocessed via the SG filter as the baseline. Four feature wavelength extraction algorithms—Lasso, Genetic Algorithm (GA), SPA, and RF—were employed to isolate optimal feature wavelength subsets. By substituting the full spectrum with a parsimonious set of informative bands for model training, this approach aims to accelerate inference time for TVB-N and TVC prediction while simultaneously mitigating computational costs.

Table 2 presents the predictive outcomes of the Transformer model optimized via four distinct feature wavelength extraction algorithms—Lasso, GA, SPA, and RF—for salmon TVB-N and TVC content. Relative to the full-spectrum modeling baseline (TVB-N prediction set R^2^ = 0.9716, RMSE = 3.0113; TVC prediction set R^2^ = 0.9721, RMSE = 0.3306), the simplified models constructed via these four dimensionality reduction techniques exhibited a marginal attenuation in accuracy but collectively maintained a robust predictive capability.

Among the evaluated methodologies, the SPA algorithm demonstrated superior feature extraction efficacy. Specifically, for TVB-N, the SPA-Transformer model achieved a prediction set R^2^ of 0.9518, outperforming the Lasso (0.9360), RF (0.9009), and GA (0.8858) models. Similarly, for TVC, the SPA method yielded the optimal results, with a prediction set R^2^ of 0.9499 and an RMSE of 0.4032, which were also significantly superior to the other three methods (Lasso R^2^ = 0.9412, RF R^2^ = 0.9089, GA R^2^ = 0.8906). In contrast, the GA algorithm exhibited relatively inferior performance across both indices, yielding prediction set R^2^ values of merely 0.8858 and 0.8906 for TVB-N and TVC, respectively.

**Figure 6:**
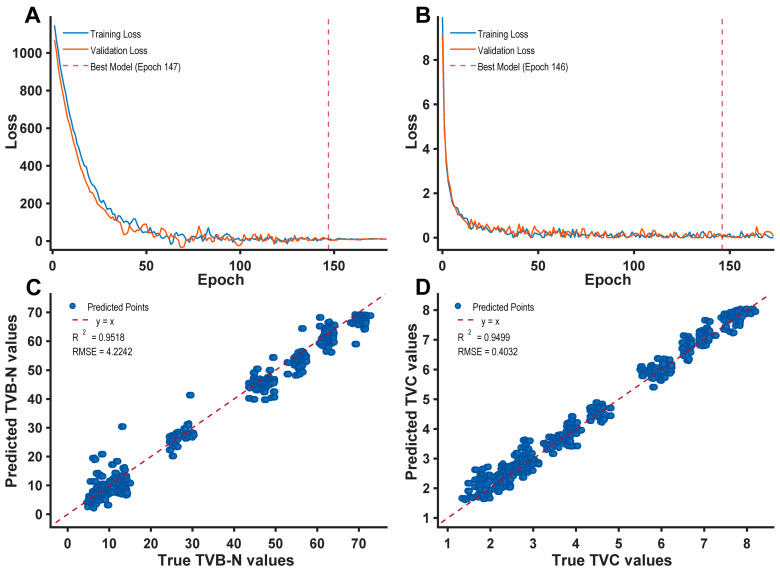
Prediction results using SG filter + Transformer model after SPA simplification. (**A**) Training and validation loss for TVB-N. (**B**) Training and validation loss for TVC. (**C**) Predicted vs. true TVB-N values. (**D**) Predicted vs. true TVC values.

Following feature wavelength selection via the SPA method (Figure 6), 30 critical wavelengths were retained for both the TVB-N and TVC prediction models. The identified set of characteristic wavelengths is defined as: Λ_SPA_ = {372.66, 387.13, 394.37, 398.00, 401.63, 405.26, 412.52, 419.80, 434.38, 449.00, 463.65, 504.14, 541.19, 556.07, 574.73, 589.70, 600.95, 634.81, 722.21, 841.92, 912.44, 936.11, 955.91, 971.78, 983.71, 995.66, 1003.63, 1011.62, 1023.62, 1039.65} nm. This consistent wavelength selection for both indicators suggests a strong intrinsic coupling between the spectral signals and the biochemical degradation processes of Atlantic salmon muscle tissue. The SG filter + Transformer model, constructed upon this reduced feature set, displayed exceptional predictive performance: the Coefficient of Determination (R^2^) for the TVB-N prediction set reached 0.9518 with an RMSE of 4.2242, while the TVC prediction set achieved an R^2^ of 0.9499 with an RMSE of 0.4032.

Compared to the full-spectrum model, the SPA-simplified model incurred only a negligible decrement in predictive precision (approximately 0.05). However, the SPA-simplified model achieved a significant enhancement in computational efficiency. To ensure a rigorous and reproducible benchmark, absolute time-consuming data were recorded across the entire computational pipeline—encompassing training, validation, and prediction for all 6342 patches under identical hardware and software configurations. Statistical evaluation revealed that the dimensionality-reduced model exhibited a training time approximately six times shorter than that of the full-spectrum baseline. Specifically, the total process time for the full-dimensional model was 74 h for the TVB-N index and 56 h for the TVC index; following SPA-based dimensionality reduction, the total time for both indices was optimized to approximately 10 h. This results in an approximate 6-fold improvement in comprehensive computational efficiency. Additionally, the inference latency was similarly reduced, enabling more rapid throughput. Crucially, while the Transformer architecture entails a higher computational overhead during the offline training phase compared to traditional linear models, its online predictive inference for a single salmon sample can be completed within 1.2 s on a standard workstation. This rapid execution demonstrates that the model effectively circumvents computational bottlenecks in practical production, fully satisfying the real-time requirements of high-throughput industrial inspection lines. To further evaluate the framework’s reliability for industrial regulatory purposes, we conducted a localized error analysis near critical legal limits. For TVB-N, the prediction set exhibited a Mean Relative Error (MRE) of 7.84% within the threshold interval (20–30 mg/100 g), ensuring high sensitivity near the 25 mg/100 g rejection limit. Similarly, for TVC, the MRE remained below 6.15% in the proximity of the 4.67 lg CFU/g and 5.0 lg CFU/g standards. These results, visualized in the clustered scatter plots (Figure 6C,D), substantiate that the model effectively sustains regulatory-grade precision while drastically reducing computational burden.

## 4. Discussion

### 4.1. Co-Evolution of TVB-N and TVC and Spectral Correlation

Stratified Pearson correlation analysis substantiated a highly significant positive correlation ($R^{2}$ > 0.90, *p* < 0.0001, n = 480) between TVB-N and TVC values, irrespective of thermal conditions. The stability of this correlation across different temperatures confirms that the spectral model captures biochemical alterations inextricably linked to bacterial population density, rather than artifacts of storage time. Given that the spatial resolution of hyperspectral imaging (at the micrometer scale) precludes the direct resolution of individual bacterial cells, the system nevertheless retains the capacity to acutely detect chemical bond vibrational signals induced by bacterial metabolites (specifically TVB-N). Consequently, the high-precision prediction of TVC achieved by the spectral model (R^2^ = 0.9721) constitutes, in essence, an “indirect determination” predicated on collinear relationships: the model captures alterations in the biochemical matrix—such as protein structural disintegration and metabolite accumulation—that are inextricably linked to bacterial population density [25].

It is noteworthy that the Transformer model exhibited marginally superior performance in predicting TVC compared to TVB-N. This implies that the spectral data encode multi-dimensional information closely associated with microbial proliferation that is not fully encapsulated by TVB-N measurements alone; such information may include alterations in muscle texture (scattering properties) induced by bacterial extracellular protease secretion, or specific spectral signatures characteristic of early-stage biofilm formation [26]. This capacity for multi-dimensional information retrieval underscores the superiority of hyperspectral technology over single-index chemical assays.

### 4.2. Analysis of Spectral Response Mechanisms (400–1000 nm)

The inherent “black box” nature of hyperspectral imaging technology has historically constrained its mechanistic interpretability. To validate the model’s reliability, this study established a physical correspondence between the feature wavelengths extracted by the model and the vibrational modes of Atlantic salmon muscle molecules. The investigated spectral range (400–1000 nm) encompasses both the visible (VIS) and short-wave near-infrared (SW-NIR) regions, which respectively harbor critical information regarding pigment degradation and the overtone absorption of chemical bonds.

### 4.3. Visible Region (400–700 nm): Pigment Degradation and Heme Oxidation

As storage time extended, the reflectance in the 600–700 nm band exhibited a marked downward trend, with the magnitude of change in the 8 °C group being significantly greater than that in the 4 °C group. This phenomenon elucidates the redox kinetics of chromophores:

Heme Protein Oxidation: The coloration of Atlantic salmon originates primarily from astaxanthin and myoglobin/hemoglobin. In the fresh state, hemoglobin exists predominantly in the oxygenated form (HbO_2_, Fe^2+^), characterized by spectral features at 540 nm and 576 nm [27]. During storage, ferrous iron oxidizes to ferric iron, forming methemoglobin and ultimately converting to brown hemichrome. Literature confirms that absorbance variations near 630–640 nm act as specific markers for methemoglobin generation [12]. The Transformer model leverages this oxidation process as an indirect feature for freshness discrimination.

Optical Masking Effect: Although astaxanthin remains chemically relatively stable, the disintegration of the myofibrillar architecture caused by collagen proteolysis alters the mean free path of photon propagation within the tissue. As “drip loss” intensifies, changes in the tissue surface refractive index induce a baseline shift in the reflectance spectrum and a “blunting” of characteristic peaks [28]. The superposition of these physical structural changes and chemical pigment alterations enriches the information entropy of the visible region.

### 4.4. Short-Wave Near-Infrared Region (700–1000 nm): Overtone and Combination Absorption of Functional Groups

The 700–1000 nm region serves as a “fingerprint zone” for the overtone absorption of hydrogen-containing groups (C-H, O-H, N-H). The accumulation of TVB-N essentially represents an increase in amines (containing N-H bonds), providing a direct basis for spectral detection.

N-H Bond Vibrational Response: The third overtone stretching vibration of N-H bonds in ammonia and amines is located at 760–800 nm, while the second overtone is found at 980–1020 nm [29]. Although the concentration of free amines is low, the degradation of the protein backbone (amide bonds) alters the microenvironment of N-H bonds (e.g., hydrogen bond rupture), causing shifts in absorption peaks. The fact that the feature wavelengths selected by the SPA algorithm fall within this interval directly attests to the model’s capacity to capture protein degradation signals.

O-H Bond and Moisture Migration (970 nm): The second overtone stretching vibration of O-H bonds in water (~70%) forms a strong absorption band at 960–980 nm [30]. A decline in freshness is accompanied by reduced water holding capacity (WHC); the outward migration of intracellular water results in drastic changes in water status (the ratio of free to bound water). Through its self-attention mechanism, the Transformer model effectively decouples weak amine signals from the strong water background and utilizes the strong collinearity between moisture migration and spoilage to aid prediction.

C-H Bond and Lipid Oxidation (930 nm): The third overtone of C-H bonds in unsaturated fatty acids is located near 930 nm [29]. Hydroperoxides and aldehydes/ketones produced by lipid oxidation induce fine spectral variations in this band. Given the spatiotemporal parallelism between lipid oxidation and protein spoilage, the deep learning model exploits this “multi-component synergistic effect” to correct prediction bias, thereby enhancing the overall robustness of the model.

### 4.5. Theoretical Limitations of Linear and Locally Constrained Modeling

The identified performance gap between the Transformer and traditional baselines highlights the inherent limitations of established spectral analysis paradigms when applied to *Salmo salar*. Traditional models like PLSR are predicated on the Beer-Lambert Law, which assumes a linear relationship between absorbance and concentration. However, in turbid biological matrices, light is subjected to stochastic multiple scattering and complex component interactions, such as moisture interference, which render the spectral response highly non-linear. Furthermore, locally constrained architectures like CNNs are restricted by their local receptive fields, failing to resolve the high-dimensional variance inherent in spoilage-related spectral data. This analysis underscores that the failure of these lightweight models is not merely a lack of precision but a fundamental structural inability to capture complex biochemical signatures.

### 4.6. Architectural Advantages of the Transformer: Long-Range Dependencies and Global Attention

In contrast to the aforementioned limitations, the Transformer architecture aligns precisely with the requirements of spectral sequence analysis. Its core superiority stems from the self-attention mechanism, which enables the computation of correlation weights between any two distal wavelength points. This allows the model to identify intrinsic stoichiometric associations—for instance, between amine signals and moisture-driven scattering shifts—that remain unresolved by traditional kernels. By utilizing a global receptive field, the Transformer integrates “chromatic variations” and “chemical bond vibrational changes” from the very first layer. This ability to decouple features within distinct subspaces allows for a synergistic correction of prediction bias, providing a robust theoretical basis for high-precision freshness monitoring.

The quantitative superiority of the Transformer ($R^{2}=0.9716$) over linear (PLSR, $R^{2}=0.837$) and locally constrained models confirms our core hypothesis: spoilage in *Salmo salar* manifests as non-linear patterns with significant long-range dependencies. This alignment between the mathematical attention mechanism and the biochemical “molecular fingerprint” provides a rigorous evidence-based confirmation of the proposed framework’s theoretical pillar.

Originally developed for NLP, the architectural nature of the Transformer perfectly aligns with the requirements of spectral sequence analysis:

Self-Attention Mechanism: This mechanism allows the model to compute correlation weights between any two wavelength points within the sequence [31]. Global Receptive Field: The Transformer possesses a global receptive field. This enables it to capture long-range interactions across the full spectrum (VIS and NIR) within the very first layer [32]. It effectively dynamically weights “chromatic variations” against “chemical bond vibrational changes,” constructing a more robust prediction model.

Combined with SG smoothing for denoising, the Transformer utilizes Multi-Head Attention to decouple features within distinct subspaces. Specific “heads” can focus on chemical absorption peaks, while others simulate and subtract complex scattering baseline backgrounds, demonstrating a feature decoupling capability that traditional algorithms lack. It is pertinent to address whether the Transformer’s predictive gains justify its increased computational load compared to conventional models like SVM, RF, and MLP. In the preliminary phase of this study, benchmarks using these models yielded coefficients of determination ($R^{2}$) consistently below 0.3, Such poor fitting performance fails to capture the complex, non-linear biochemical signatures of spoilage, rendering lightweight models inadequate for high-stakes food safety applications. In contrast, the SPA-Transformer framework provides a transformative increase in precision ($R^{2}\geq 0.95$), which is essential for reducing quality misjudgment rates and mitigating economic losses in the cold chain. Given that the computational complexity is primarily localized to the one-time training phase, the substantial improvements in accuracy and stability offer a favorable trade-off, providing critical technical support for precision-driven aquatic product supply chains.

### 4.7. Efficacy Evaluation of Feature Selection Algorithms

To mitigate the “curse of dimensionality,” this study utilized SPA to extract 30 feature wavelengths, maintaining exceptionally high precision ($R^{2}=0.9518$). Compared to the state-of-the-art (SOTA), which often requires full-spectrum deep learning to achieve similar accuracy, our SPA-Transformer framework offers significant added value by achieving industrial-grade precision with only 19% of the original spectral data. While previous studies using PLSR for fish quality achieved $R^{2}$ values typically ranging from 0.80 to 0.88, our approach delivers a measurable +14% improvement in predictive stability. This performance-to-efficiency ratio establishes a new benchmark for non-destructive inspection, providing the theoretical underpinning for developing portable, cost-effective multispectral sensors for cold-chain monitoring.

The superiority of SPA selected in this experiment is evident: as a forward variable selection algorithm, SPA is designed to screen for wavelength combinations carrying the richest “independent information.” In comparison, Lasso is prone to arbitrary selection among highly correlated variables, leading to instability in the feature set, while GA are susceptible to entrapment in local optima. Furthermore, SPA compresses the input dimensions from hundreds to merely 30, enhancing computational efficiency by a factor of approximately six. This provides a rigorous theoretical basis for the design of low-cost multispectral cameras. In industrial production lines, the deployment of cameras equipped with customized filters targeting these 30 specific bands constitutes a cost-effective alternative to expensive full-spectrum hyperspectral imaging systems.

### 4.8. Limitations and Future Perspectives

While the SPA-Transformer model demonstrates high precision, its interpretative scope is bounded by several factors. First, the reliance on a single batch from Norwegian aquaculture ignores potential variability between producers and harvesting seasons. Objectively, variations in muscle lipid content and protein profiles among salmon from different geographical origins directly influence hyperspectral signatures. Second, the absence of inter-laboratory validation remains a key limitation; the current results are predicated on a standardized experimental setup with fixed lighting (45° angle) and constant focal distances (58 cm). In real industry conditions, fluctuations in ambient lighting and conveyor belt vibrations pose significant threats to spectral stability. Furthermore, while we modeled spoilage at 4 °C and 8 °C to simulate commercial scenarios, the model’s performance under extreme thermal abuse or during the “thawing-refreezing” cycle—common in global logistics—has not yet been evaluated. To transition toward a generalized industrial tool, future work must incorporate diverse fish species, irregular anatomical cuts, and samples with varying fat-to-protein ratios.

### 4.9. Validity of Patch-Based Data Partitioning

A critical consideration in patch-based learning is the risk of pseudo-replication—where highly correlated patches from the same fillet might artificially inflate performance metrics. However, we argue that our strategy is justified by the inherent spatial heterogeneity of *Salmo salar* muscle tissue. Each non-overlapping patch represents a distinct biochemical micro-environment, capturing localized variations in fiber density and moisture distribution that global average spectra might overlook. By integrating patches from multiple orientations alongside those from 480 independent biological individuals, the dataset encompasses both intra-fillet and inter-fillet variances. This approach forces the Transformer to learn generalized spoilage patterns robust to localized spectral fluctuations, although we acknowledge that future inter-individual validation on a per-fillet basis is necessary to fully mitigate pseudo-replication risks. However, we explicitly acknowledge that using multiple patches from a single fillet introduces a risk of internal data dependence, as these patches share the same physiological history and global labels. This potential for pseudo-replication remains a current limitation, as the high correlation between neighboring patches may lead to an overestimation of the model’s local sensitivity. In high-throughput inspection lines, localized tissue heterogeneity and individual biological differences occur simultaneously. By utilizing 960 images obtained through bilateral scanning of 480 independent samples, the dataset provides a holistic representation of these coexisting error sources. Evaluating the framework’s accuracy against this integrated variance provides a more authentic measure of its comprehensive predictive stability and practical suitability for commercial deployment, as opposed to an idealized separation of error components.

## 5. Conclusions

This study centered on the application of Hyperspectral Imaging (HSI) technology for the prediction of TVB-N and TVC values in Atlantic salmon. Through the systematic optimization of preprocessing methodologies and modeling strategies, the following principal achievements were realized:

In the screening of preprocessing protocols, comparative validation identified the Savitzky-Golay (SG) filter as the superior method. It demonstrated exceptional efficacy in attenuating spectral noise while preserving critical feature fidelity, thereby providing a high-quality data foundation for subsequent modeling.

Regarding model architecture, the synergistic integration of SG smoothing with the Transformer model emerged as the definitive solution, yielding the highest predictive accuracy. Specifically, the prediction set for TVB-N achieved a Coefficient of Determination (R^2^) of 0.9716 with a Root Mean Square Error (RMSE) of 3.0113, while the prediction set for TVC attained an R^2^ of 0.9721 and an RMSE of 0.3306.

To further reconcile the trade-off between predictive precision and computational efficiency, the Successive Projections Algorithm (SPA) was employed to distill 30 characteristic wavelengths.

The optimized model maintained robust performance, achieving an R^2^ of 0.9518 (RMSE = 4.2242) for the TVB-N prediction set and an R^2^ of 0.9499 (RMSE = 0.4032) for the TVC prediction set. Furthermore, a cost-benefit analysis confirms that the marginal increase in training complexity is far outweighed by the significant boost in prediction accuracy compared to conventional lightweight regressors. The streamlined architecture, achieving near-instantaneous inference (within seconds), bridges the gap between sophisticated deep learning and the practical constraints of real-time monitoring.

By systematically curating spectral preprocessing techniques, modeling algorithms, and feature wavelength extraction methods, combined with rigorous parameter tuning, a high-precision quality prediction model for salmon was successfully established. The proposed “HSI + SG + SPA + Transformer” framework holds significant pragmatic value for the intelligent evolution of the food industry. By reducing the required spectral data by approximately 81% through SPA without compromising precision, this framework facilitates a transition from traditional destructive sampling to comprehensive on-line non-destructive monitoring. This technology provides the theoretical foundation for developing portable, cost-effective multispectral sensors targeting the 30 identified characteristic wavelengths, which is a key requirement for commercial implementation in resource-constrained logistics. Furthermore, with a near-instantaneous inference time of 1.2 s, the model effectively meets the real-time throughput demands of industrial inspection lines. Such an integrated predictive tool is essential for reducing quality misjudgment rates and mitigating economic losses within the global salmon cold chain. Future research will focus on validating this framework across larger, multi-batch datasets and extreme thermal conditions to ensure its comprehensive stability across diverse commercial environments.

## Figures and Tables

**Table 1:** Prediction Results of TVB-N and TVC by Different Models under Various Preprocessing Conditions.

Metric	Pre-Processing Method	Model	Training Set		Validation Set		Predicion Set	
			R^2^	RMSE	R^2^	RMSE	R^2^	RMSE
TVB-N	Raw	Transformer	0.9928	2.0466	0.9561	4.0644	0.9475	4.4780
	SG filter		0.9869	2.7749	0.9728	2.0275	0.9716	3.0113
	SNV		0.9941	1.8630	0.9571	3.5017	0.9497	4.3610
	Baseline Correction		0.9861	2.8534	0.9407	4.8828	0.9464	4.5326
	Min-Max Normalization		0.9712	4.1054	0.9017	7.5749	0.8767	8.3938
	Raw	PLSR	0.916	6.842	0.862	8.779	0.820	9.287
	SG filter		0.909	7.095	0.870	8.496	0.837	8.843
	SNV		0.910	7.056	0.789	10.850	0.788	10.063
	Baseline Correction		0.909	7.115	0.839	9.457	0.788	10.063
	Min-Max Normalization		0.905	7.271	0.819	10.040	0.809	9.546
	Raw	MLR	0.920	6.663	0.850	9.149	0.820	9.288
	SG filter		0.920	6.662	0.850	9.149	0.819	9.293
	SNV		0.910	7.054	0.789	10.851	0.790	10.033
	Baseline Correction		0.915	6.889	0.823	9.940	0.794	9.921
	Min-Max Normalization		0.914	6.918	0.796	10.662	0.805	9.652
TVC	Raw	Transformer	0.9914	0.1864	0.9540	0.4278	0.9639	0.3783
	SG filter		0.9871	0.2277	0.9735	0.3254	0.9721	0.3306
	SNV		0.9929	0.1685	0.9695	0.3486	0.9688	0.3515
	Baseline Correction		0.9825	0.2652	0.9400	0.4888	0.9385	0.4940
	Min-Max Normalization		0.9897	0.2030	0.9483	0.4537	0.9466	0.4603
	Raw	PLSR	0.917	0.572	0.874	0.705	0.835	0.761
	SG filter		0.909	0.600	0.879	0.690	0.837	0.755
	SNV		0.912	0.588	0.807	0.872	0.813	0.810
	Baseline Correction		0.915	0.580	0.856	0.754	0.810	0.816
	Min-Max Normalization		0.907	0.771	0.832	0.812	0.831	0.771
	Raw	MLR	0.922	0.554	0.864	0.733	0.837	0.755
	SG filter		0.922	0.554	0.864	0.732	0.837	0.755
	SNV		0.913	0.585	0.806	0.873	0.815	0.806
	Baseline Correction		0.918	0.570	0.841	0.791	0.813	0.809
	Min-Max Normalization		0.916	0.577	0.811	0.862	0.830	0.772

**Table 2:** Prediction Results of TVB-N and TVC by the Transformer Model under Different Feature-Wavelength Extraction Methods.

Metric	Feature-Wavelength Extraction Method	Model	Training Set		Validation Set		Prediction Set	
			R^2^	RMSE	R^2^	RMSE	R^2^	RMSE
TVB-N	Lasso	Transformer	0.9763	3.7261	0.9600	4.0810	0.9360	3.5185
	GA		0.9508	5.3688	0.8897	7.1047	0.8858	5.0395
	SPA		0.9864	2.8259	0.9423	3.8644	0.9518	4.2242
	RF		0.9451	4.0730	0.9143	4.3439	0.9009	4.4902
TVC	Lasso	Transformer	0.9631	0.3851	0.9245	0.4490	0.9412	0.4499
	GA		0.9431	0.3781	0.8713	0.4168	0.8906	0.4546
	SPA		0.9795	0.2869	0.9472	0.4090	0.9499	0.4032
	RF		0.9729	0.3302	0.9143	0.4848	0.9089	0.4973

## Data Availability

The original contributions presented in this study are included in the article. Further inquiries can be directed to the corresponding author.
